# Lipid and Lipoprotein Metabolism in Microglia

**DOI:** 10.3389/fphys.2020.00393

**Published:** 2020-04-28

**Authors:** Bailey A. Loving, Kimberley D. Bruce

**Affiliations:** ^1^School of Medicine, University of Colorado, Anschutz Medical Campus, Aurora, CO, United States; ^2^Division of Endocrinology, Metabolism and Diabetes, University of Colorado, Anschutz Medical Campus, Aurora, CO, United States

**Keywords:** lipid and lipoprotein metabolism, microglia, neurodegenerating diseases, Alzheimer’s disease, lipid, lipoprotein, multiple scleorsis (MS), APOE

## Abstract

Microglia, once viewed as static bystanders with limited homeostatic functions, are now considered key players in the development of neuroinflammatory and neurodegenerative diseases. Microglial activation is a salient feature of neuroinflammation involving a dynamic process that generates multitudinous microglial phenotypes that can respond to a variety of situational cues in the central nervous system. Recently, a flurry of single cell RNA-sequencing studies have defined microglial phenotypes in unprecedented detail, and have highlighted robust changes in the expression of genes involved in lipid and lipoprotein metabolism. Increased expression of genes such as Apolipoprotein E (ApoE), Triggering Receptor Expressed on Myeloid Cells 2 (TREM2) and Lipoprotein Lipase (LPL) in microglia during development, damage, and disease, suggest that increased lipid metabolism is needed to fuel protective cellular functions such as phagocytosis. This review describes our current understanding of lipid and lipoprotein metabolism in microglia, and highlights microglial lipid metabolism as a modifiable target for the treatment of neurodegenerative diseases such as Alzheimer’s disease and multiple sclerosis.

## Introduction

Microglia are functionally distinct brain-resident macrophages that are seeded developmentally and maintained by self-proliferation (Ajami et al., 2007; Alliot et al., 1991; Askew et al., 2017). Once considered static bystanders with limited homeostatic functions, it is now becoming increasingly clear that microglia interact with all CNS components and have a marked impact on brain health and disease (Li and Barres, 2018). Microglia are highly plastic and respond to a variety of environmental cues by switching to appropriate activation states. While some activation states are adaptive and contribute to homeostatic functions, others are maladaptive and associated with neuroinflammation. Microglial activation and dysfunction are salient features of neuroinflammatory and neurodegenerative diseases (NDs), such as Alzheimer’s disease (AD), Parkinson’s disease (PD), and multiple sclerosis (MS) (Colonna and Butovsky, 2017). Thus, it has been increasingly recognized that understanding the heterogeneity of microglial activation in the context of disease may facilitate the design of therapeutics that dampen the detrimental effects of microglial activation, while augmenting the beneficial effects of “alternatively” activated microglia (Li and Barres, 2018).

Recently, several comprehensive single-cell RNA-sequencing (scRNAseq) analyses of microglia *ex vivo* have defined the transcriptomic identities of microglia with temporal (Grabert et al., 2016; Hammond et al., 2019; Li et al., 2019), regional (Grabert et al., 2016; Hammond et al., 2019; Li et al., 2019), and disease state specificity (Hammond et al., 2019; Keren-Shaul et al., 2017; Mathys et al., 2017). Various clusters of microglia with similar gene expression profiles have been mapped to specific regions and developmental or disease stages. A striking feature of these studies is the identification of microglial clusters with common metabolic characteristics. For example, Keren-Shaul et al. (2017) used the 5XFAD murine model of AD to define Disease Associated Microglia (DAMs) that express a distinct set of genes associated with lipid and lipoprotein metabolism (e.g., Apolipoprotein E [ApoE], Lipoprotein Lipase [LPL], and Triggering Receptor Expressed On Myeloid Cells 2 [TREM2]) (Keren-Shaul et al., 2017). This transcriptional signature represents a preference for lipids as fuel substrates that fulfill the greater bioenergetics needs of activated microglia (Keren-Shaul et al., 2017). Interestingly, these DAMs bear a striking resemblance to microglial clusters observed during early postnatal life (P4/P5) (Hammond et al., 2019), and in proliferative-region-associated-microglia (PAMs), which reside in regions of the early postnatal brain with active gliogenesis and neurogenesis (Li et al., 2019). A similar signature is also observed in microglia of the demyelinating brain (Hammond et al., 2019), and in the later stages of a CK-p25 murine model of DNA damage and neurodegeneration (Mathys et al., 2017).

Even though these studies have varied in their experimental approach, they have repeatedly implicated consistent changes in microglial metabolism during microglial activation. These studies have reignited our interest in “immunometabolism,” particularly in the context of neurodegenerative disease (ND). Here, we review our current understanding of lipid and lipoprotein metabolism in microglia to identify potentially targetable metabolic processes, which may ameliorate detrimental microglial responses and help develop novel therapeutic interventions for ND and beyond.

## Importance of Microglia in Brain Health and Disease

### Development

Microglia represent 5 to 15% of the adult CNS cell population and constitute the largest population of immune cells in the brain. They play a major role in the development of the CNS, maintain homeostasis within the healthy brain, and can initiate, propagate, and/or resolve inflammatory responses. To understand how these cells are capable of such feats, it is important to understand the origin and cell lineage of microglia. Microglia originate from yolk sac erythromyeloid precursor cells in mice at embryonic day 7.5 (E7.5) (Alliot et al., 1991). These cells infiltrate both the neuro-epithelium and cephalic mesenchyme at days E8.5/E9.0 and undergo dramatic expansion until the second postnatal week (Alliot et al., 1991, 1999; Tambuyzer et al., 2009; Ginhoux et al., 2010; Mizutani et al., 2012; Crotti and Ransohoff, 2016). Infiltration into the CNS is not uniform, and location-dependent heterogeneity in density, morphology, and gene expression is observed (Lawson et al., 1990). Using scRNA-seq in conjunction with RNA immunohistochemistry (IHC) *in situ* across developmental time points, microglial heterogeneity within parenchyma, (PAM), and meninges, border-associated microglia (BAM), is now mapped into specific subpopulations by gene cluster (Goldmann et al., 2016; Prinz et al., 2017; Li et al., 2019; Ochocka et al., 2019; Van Hove et al., 2019). Although microglial ontogeny is mostly preserved across subpopulations, the region of the brain in which the microglia reside significantly affects the phenotype and response to their microenvironment (Grabert et al., 2016). At E9.5, yolk sac progenitors develop into embryonic microglia (Rosenbauer and Tenen, 2007). These embryonic microglia are a distinct tissue specific macrophage population, which is different from circulating myeloid cells. Specifically, erythromyeloid precursor cells develop into CD45^+^, c-kit^*lo*^, C-X3-C Motif Chemokine Receptor 1 (CX3CR1)immature cells, when in turn mature into CD45^+^, c-kit^–^, CX3CR1^+^, which invade the brain using specific metalloproteases (Kierdorf et al., 2013). Following infiltration, factors such as Transforming growth factor β (TGFβ), IL-34 and Colony stimulating factor 1 receptor (CSF-1R) are necessary for the terminal differentiation of resident microglia (Butovsky et al., 2014). Although it remains somewhat controversial, recent studies suggest that in addition to canonical, non-*Hoxb8* expressing microglia that infiltrate the brain at E9.5, there is a “second wave” of yolk-sac progenitors that are greatly expanded in the fetal liver prior to infiltrating the brain at E12.5 (De et al., 2018). Importantly, this second wave of cells may give rise to microglia-like cells, with distinct characteristics such as expression of Membrane-spanning 4-domains subfamily A member 7 (Ms4a7) and ApoE (Bennett and Bennett, 2020).

At E11/E16, neural stem cells (NSCs) at the dorsoventral boundary begin a massive expansion and differentiate into neural progenitor cells (NPCs), oligodendrocyte precursor cells (OPCs), and mature oligodendrocytes (OLs) (Naruse et al., 2017). Over half of these mature OLs eventually undergo apoptosis (Barres et al., 1992). Many of these mature apoptotic OLs are phagocytosed by an early phagocytic subset of PAM (Li et al., 2019), highlighting the role of microglia in primary myelination. In addition, a subtype of microglia associated with white matter and axon tracts express high levels of LPL and Secreted Phosphoprotein 1 (Spp1) (Hammond et al., 2019), which could potentially facilitate clearance of myelin-derived lipid debris. Chemokines such as chemokine (C-X-C motif) ligand 1 (CXCL1), expressed in high quantities by neurons, stimulate microglial migration (Harrison et al., 1998; Jakovcevski et al., 2009), potentially to sites with active myelinogenesis. It is thought that microglia regulate primary myelinogenesis through a critical source of neuroprotective Insulin growth factor 1 (IGF1), known to regulate OL function (Wlodarczyk et al., 2017). Mature OLs are lipid-rich, requiring cholesterol and both essential and non-essential fatty acids (FAs) to effectively produce myelin (Mathews and Appel, 2016; Dimas et al., 2019). Although astrocytes provide OLs with cholesterol via effluxed lipoprotein particles, OLs can also produce cholesterol *de novo* via Phosphoinositide 3-kinase/Protein kinase B/mammalian target of rapamycin (PI3K)/(Akt)/(mTOR) signaling (Chen et al., 2013; Mathews and Appel, 2016). The precise role of microglia in OL lipid metabolism is unclear, however, studies outlined above suggest a key role in the phagocytosis of lipid-rich debris from myelin and apoptosed OPCs and OLs.

While recent studies have shown that microglia are extremely diverse during early development, it is clear that they become less heterogeneous during adulthood (Hammond et al., 2019). Nonetheless, the dynamic transcriptional signatures of microglia exposed to injury, aging or disease, suggest that the adult microglia are readily poised to adapt to challenges in the local environment.

### Synaptic Pruning

It has been previously observed in healthy brain that so-called “resting microglia” are in fact very active. They are highly mobile and take, on average, a 5 min sample of one neuronal synapse per hour (Davalos et al., 2005; Nimmerjahn et al., 2005; Wake et al., 2009). Additionally, synapses in ischemic areas are turned over following microglial detection (Wake et al., 2009). This sampling of synapses hints at the possibility that microglia are involved in the crucial process of synaptic pruning. The expression of the chemokine fractalkine (CX3CL1), a strong microglial chemoattractant and whose receptor in the CNS is expressed solely in microglia, is up-regulated in neurons during development (Harrison et al., 1998; Jung et al., 2000; Cardona et al., 2006; Liang et al., 2009). Through GFP-labeling of microglia and immunohistochemistry against postsynaptic density protein 95 (PSD95), a marker of postsynaptic density, stimulated emission depletion (STED) microscopy revealed colocalization of PSD95 and GFP. Moreover, electron microscopy of these microglia show the presence of both clathrin-coated and non-clathrin-coated vesicles containing PSD95 as well as Synaptosome Associated Protein 25 (SNAP25), a marker of presynaptic density (Paolicelli et al., 2011). These data strongly suggest that microglia are actively involved in synaptic pruning. A closer look at hippocampal synapses via light sheet fluorescence microscopy and 3D ultrastructural characterization using Correlative light and electron microscopy (CLEM) found that microglia do not directly phagocytose entire synapses (Weinhard et al., 2018). Instead, they trogocytose—selective partial phagocytosis—the membranes of presynaptic boutons and axons without any evidence for elimination of dendritic spines (Weinhard et al., 2018). The exact mechanism of trogocytosis has yet to be elucidated.

### Phagocytosis

Microglia play an important role as the immune effector cells of the central nervous system. In line with this role, microglia express many cell surface factors that are also expressed by peripheral myeloid cells and macrophages such as integrin’s, toll-like receptors, scavenger receptors, and TREM2 (Aldana, 2019). Although these similarities have made the isolated study of microglia somewhat challenging, there has been a concerted effort to define the characteristics that set microglia apart from other myeloid cells. For example, while microglia and bone marrow derived macrophages both express CD11B and CD45 (although high for macrophages and low for microglia), microglia express higher levels of Transmembrane Protein 119 (Tmem119), CX3CR1 and Solute Carrier Family 2 Member 5 (Slc2a5) (Haage et al., 2019). It is likely that this differential expression represents the homeostatic functions that are specific to microglia and not peripheral cells such as synaptic pruning, neuronal survival, and synaptogenesis (Bilimoria and Stevens, 2015). Such neuroprotective functions are largely due to the phagocytic nature of microglia (Janda et al., 2018). Microglia are highly efficient phagocytes that remove apoptotic or necrotic cells (Green et al., 2016), and unfolded proteins such as amyloid beta (Aβ) or neuromelanin. Engulfment of myelin debris is also a key function of microglial sub-populations associated with remyelination and repair (Olah et al., 2012). Furthermore, microglia are an important part of the innate immune system and are activated in response to infections in order to directly phagocytose potentially pathogenic microorganisms (Nau et al., 2014). Microglial phagocytosis initiates the adaptive arm of the immune system via antigen presentation (Litman et al., 2005). Overall, the immune functions of microglia showcase their plasticity and ability to respond to a wide variety of stimuli. However, the response can sometimes become chronic and maladaptive. This prolonged microglial activation is a salient feature of many NDs such as AD, Parkinson’s disease and MS.

### Disease

#### Metabolic Disorders

Metabolic disorders such as obesity have implicated in the pathological activation of microglia largely due to an increase in systemic inflammation. For example, the leptin deficient mouse model of type 2 diabetes (db/db), shows increased inflammatory chemokine and cytokine expression in the CNS (Kumar et al., 2014). In addition, several studies have shown that exposure to a high-fat diet (HFD) can increase microglial activation, even without peripheral inflammation (Ziko et al., 2014; Kang et al., 2016). In fact, recent work as shown that only 3 days of HFD exposure is sufficient to promote gliosis in the hypothalamus (Thaler et al., 2012). Since microglia are seeded developmentally and are particularly long-lived, they are a potential conduit for the transmission of “developmentally programmed” dietary exposures. In support of this hypothesis, microglia in the paraventricular nucleus of the hypothalamus can be programmed to an active state following HFD exposure during very early life; a phenotype that persists into adulthood (Morari et al., 2014). It is likely that circulating inflammatory mediators and lipids and lipoproteins have greater penetrance to the hypothalamus than other regions of the brain, which may explain the rapid metabolic polarization of microglia in this region.

#### Schizophrenia

Dysregulation of synaptic pruning has been increasingly implicated in the pathophysiology of both NDs and psychiatric disorders. In schizophrenia (SZ), patients have decreased gray matter thickness and reduced overall brain volume (Ziermans et al., 2012; Cannon et al., 2015). This is correlative with a decrease in synaptic density (Glantz and Lewis, 2000; Glausier and Lewis, 2013). To study this phenomenon, peripheral monocytes were induced into a validated microglia-like (iMG) cell culture model (Sellgren et al., 2019). Interestingly, iMGs from patients with SZ that are co-cultured with neurons display elevated internalization of PSD95 and SNAP25 as well as a utilization of the complement system, suggesting that microglial synaptic pruning is increased in patients with ongoing SZ (Sellgren et al., 2019). Microglia-mediated degradation of synapses is also seen in AD (Lacor et al., 2004; Chu et al., 2010; Schafer et al., 2012; Bialas and Stevens, 2013; Sekar et al., 2016; Panayiotou et al., 2017; Shi et al., 2017).

#### Alzheimer’s Disease

Alzheimer’s Disease is the most common cause of dementia in the elderly, characterized by gradual memory loss and cognitive decline. The first phase of the disease involves the subclinical, gradual buildup of extra-cellular monomeric Aβ (mAβ) that coalesces into oligomers and eventually larger amyloid fibrils/plaques. This leads to the clinically significant second phase of the disease, involving the formation of hyperphosphorylated tau neurofibrillary tangles that are associated and the destruction of neurons; the hallmark of AD. Although cognitive decline occurs most drastically during the second phase, the gradual phase has also been implicated in the deterioration of synaptic density as a result of increased microglia-mediated synapse loss (Carroll, 2004; Gasque, 2004; Reichwald et al., 2009; Ransohoff, 2016). This is primarily facilitated by fibrillar Aβ (fAβ) and oligomeric Aβ (oAβ) aggregation onto neuronal post-synaptic terminals leading to complement deposition, microglial activation following synapse elimination and neural network dysfunction (Carroll, 2004; Gasque, 2004; Reichwald et al., 2009; Pan et al., 2011; Ransohoff, 2016). C3 receptor (CD11b/CD18) wielding microglia are the key mediators of Aβ clearance and show a differential phagocytic response depending on the morphology of the encountered Aβ (Carroll, 2004; Gasque, 2004; Reichwald et al., 2009; Ransohoff, 2016). Microglial exposure to fAβ induces a classical phagocytic response, which may help to facilitate the removal of fAβ and prevent plaque formation (Pan et al., 2011). In support, recent scRNA-seq studies in the 5XFAD model of AD have shown a profound increase in DAM, which have elevated expression of factors involved in lipid uptake and phagocytosis, likely in an attempt to clear fAβ at later stages of the disease (Keren-Shaul et al., 2017). Although the role of microglia in AD pathogenesis is a research-intensive area, whether specific microglial subpopulations contribute or prevent AD progression remains to be empirically determined.

**FIGURE 1:**
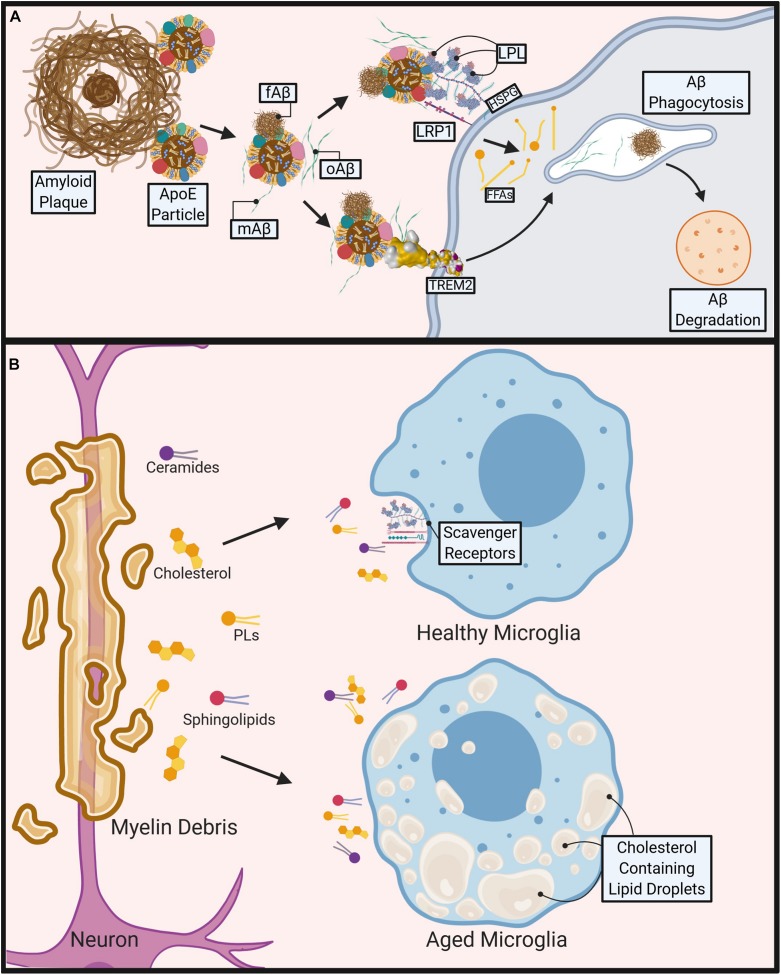
Role of microglial lipid metabolism in the pathogenesis of Alzheimer’s disease (AD) and Multiple Sclerosis (MS). **(A)** During AD, Apolipoprotein E4 (ApoE4) containing particles are thought to cause polymerization of Amyloid-Beta (Aβ) contributing to fibrillar Aβ (fAβ), plaque formation and AD pathogenesis. Aβ phagocytosis/clearance may prevent plaque formation. TREM2 binds to oligomeric Aβ (oAβ) and may bind to ApoE containing lipoprotein particle-bound monomeric Aβ (mAβ) and fAβ to facilitate phagocytosis and degradation of Aβ. Lipoprotein Receptor-like protein 1 (LRP1) and Lipoprotein Lipase (LPL) tethered to the microglial cell surface via Heparan sulfate proteoglycans (HSPGs) may also bind to Aβ directly or via an interaction with Aβ-bound ApoE containing lipoproteins, to facilitate Aβ uptake and degradation. **(B)** During MS, demyelination causes release of myelin-derived lipids such as ceramides, cholesterol, phospholipids (PL), and sphingolipids. Healthy microglia clear lipids via cell surface scavenger receptors (e.g., LRP1, LPL). However, aged, or pro-inflammatory microglia accumulate cholesterol and other neutral lipids, which impairs their ability to phagocytose myelin debris, leading to reduced and abnormal remyelination. Created with Biorender.com.

The clearance of Aβ and its tendency to aggregate can be significantly altered by lipid and lipoprotein metabolism (see Figure 1A). Interestingly, Alois Alzheimer originally described an increased quantity of “lipoid granules” in the AD brain as a pathological hallmark, suggesting that irregular lipid and lipoprotein metabolism may be a driving factor (Foley, 2010). Increased free-cholesterol containing lipid rafts (Riddell et al., 2001; Ehehalt et al., 2003; Hattori et al., 2006; Marquer et al., 2011), which have been shown to increase Aβ aggregation (Wisniewski et al., 1994; Bhattacharyya and Kovacs, 2010; Bryleva et al., 2010) may be a potential underlying mechanism. Additionally, ApoE, the most abundant apolipoprotein in the CNS, is also known to modulate Aβ aggregation in an isoform dependent manner (LaDu et al., 1994). Furthermore, gangliosides (which contain ceramide) within Aβ aggregates may contribute to the persistence of amyloid plaques by evading microglial detection via their interaction with sialic acid-binding Ig-like lectin (siglec-11), a negative immune receptor (Salminen and Kaarniranta, 2009). There is an increasing focus toward microglia-mediated lipid and lipoprotein metabolism in the brain and their effects on systemic and neuroinflammatory diseases (Di Paolo and Kim, 2011; Bruce et al., 2017; Reale and Sanchez-Ramon, 2017; Xicoy et al., 2019).

#### Multiple Sclerosis

In progressive forms of MS, the progression of chronically active demyelination lesions leads to worsening disability (Absinta et al., 2019). Within these lesions, myelin, which is composed mostly of lipid, is destroyed by microglia and lipid-laden infiltrating macrophages amongst other immune cells (Raine et al., 1981). It is here that lipid metabolism is most crucial in the modulation of disease. The presence of myelin debris itself is known to cause a sustained inflammatory response (Clarner et al., 2012; Cantuti-Castelvetri et al., 2018; Kopper and Gensel, 2018). Additionally, cholesterol-breakdown products are significantly higher within the brain and CSF of these patients. 7-ketocholesterol (7KC) is among these products and is associated with mitochondrial dysfunction and attenuated lipid processing through FA oxidation (FAO) in OLs (Diestel et al., 2003; Leoni et al., 2017). In microglia, 7KC rapidly enters the nucleus and activates poly (ADP-ribose)-polymerase (PARP)-1, which induces a pro-inflammatory phenotype (Diestel et al., 2003; Kauppinen and Swanson, 2005). In some ways this is helpful, as activated microglia are more mobile and significantly increase their phagocytic activity in order to clear debris effectively (Fu et al., 2014). However, a recent study has shown that as phagocytic microglia age they lose the capacity to effectively efflux cholesterol, form intracellular cholesterol crystals, and propagate a maladaptive pro-inflammatory response (Cantuti-Castelvetri et al., 2018) (see Figure 1B). In contrast, omega-3 (n-3) polyunsaturated FAs (PUFAs) have been shown to reduce inflammation and induce an anti-inflammatory phenotype within microglia (Hopperton et al., 2016; Chen et al., 2018; Layé et al., 2018). Similarly, an increased intake of total dietary PUFAs is associated with a reduced risk of developing MS (Bjornevik et al., 2017). In addition, we have previously shown that LPL, the rate-limiting enzyme in the hydrolysis of triglyceride (TG) rich lipoproteins, is associated with an anti-inflammatory microglial phenotype due to its complex role in lipid uptake and metabolic reprogramming (Bruce et al., 2018).

## Metabolic Reprogramming in Macrophages and Microglia

It is well established that macrophages change their metabolic profile to meet the increased bioenergetic demands of activation. Although a similar “metabolic reprogramming” also occurs in microglia, our understanding of this process is less well understood. For this reason, it is useful to review the literature describing the interaction between metabolism and inflammation in macrophages to serve as a relative model for microglia. When macrophages are homeostatic, or quiescent, they catabolize various substrates (glucose, amino acids, fatty acids) to be utilized in the TCA cycle to produce electrons (carried in the form of NADH/FADH2). These electrons are then used during mitochondrial oxidative phosphorylation (OXPHOS) to drive adenosine triphosphate (ATP) production (Mehta et al., 2017). In contrast, pro-inflammatory macrophages show a metabolic shift toward glycolysis and away from OXPHOS (Van den Bossche and Saraber, 2018). The metabolic preference for energy derived from glycolysis over OXPHOS in normoxic conditions is reminiscent of the metabolic profile of many types of tumors. In these tumor cells, glycolysis predominates despite sufficient oxygen for oxidative metabolism to proceed; a phenomenon first described by Otto Warburg in 1927, thus termed the “Warburg effect” (Warburg et al., 1927). In macrophages, metabolic polarization to a Warburg-like metabolism can follow exposure to a range of stimuli, including LPS, the toll-like receptor (TLR) 3 ligand poly(I:C), and type 1 interferon (IFN) (Kelly and O’Neill, 2015). It has been suggested that activated macrophages shift toward glycolytic metabolism to preserve the macromolecules that may be needed to synthesize new proteins required for a given activation state (Rambold and Pearce, 2018). For example, glucose metabolism feeds the pentose phosphate pathway (PPP), which increases the production of purines and pyrimidines, important for biosynthesis in the activated cell. Increased flux through the PPP also provides NADPH for NADPH oxidase, which generates reactive oxygen species (ROS) that can be used as an anti-bacterial mechanism (West et al., 2011). It is also thought that the increase in glycolysis generates ATP quickly, and although inefficient in comparison to oxidative metabolism, provides the necessary energy to support cell activation in the shortest time frame. Importantly, recent studies using microglia isolated from 5XFAD mice have shown similar impairments in metabolism including shifts toward glycolysis, which can be reversed following INF-γ treatment (Baik et al., 2019). There are a number of mechanisms thought to contribute to the metabolic reprogramming of macrophages and microglia, although here we will focus on those involving alterations in lipid metabolism, there are several reviews that discuss the mechanisms in more detail (Kelly and O’Neill, 2015; Langston et al., 2017).

Macrophage lipid metabolism is profoundly altered during polarization. For example, following macrophage activation, the TCA cycle is interrupted and acetyl-CoA is shunted to the synthesis of lipid precursors for inflammatory mediators (Jha et al., 2015). Specifically, following intraperitoneal (IP) injection of LPS or zymosan (a ligand found on surface of yeast) there is a dramatic increase in the synthesis of cholesterol ester (CE) and FAs in peritoneal macrophages (Posokhova et al., 2008). Furthermore, activated macrophages take up significantly more native and acetylated low-density-lipoproteins (LDL), which contributes to CE synthesis, whereas non-acetylated LDL uptake is inhibitory to CE synthesis in control cells (Wang et al., 2007). In addition, LPS treatment leads to TG accumulation in macrophages, which is coupled with decreased FAO and TG lipolysis, without altered expression of intracellular lipases (Adipose triglyceride lipase [ATGL] and hormone sensitive lipase [HSL]) (Feingold et al., 2012). In contrast, ‘alternative activation’ of macrophages is associated with increased FAO, raising the possibility that controlling fatty acid metabolism may attenuate inappropriate activation (Namgaladze and Brune, 2016). Several targets have been identified that may control FA metabolism in the context of inflammation, with a particular emphasis on mitochondrial FAO. For example, promoting mitochondrial β-oxidation has been shown to attenuate endoplasmic reticulum (ER) stress that can be induced by the saturated fatty acid (SFA) palmitate (C16:0) (Namgaladze et al., 2014). Conversely, knockdown, or pharmacological inhibition of mitochondrial FAO leads to exacerbated ER stress and inflammation in response to palmitate (Namgaladze et al., 2014). In support, over expression of a mutant from of Carnitine Palmitoyltransferase 1A (CPT1A) that is insensitive to malonyl-CoA inhibition (and thus shows enhanced mitochondrial FAO) prevents palmitate-induced inflammation, ER stress, and oxidative damage (Malandrino et al., 2015). While there may be deleterious effects of increased FAO, particularly in tissue-specific or hypoxic conditions, overall it has been suggested that a major consequence of increased macrophage FAO is a diversion of fatty acid flux away from the formation of FA metabolites that cause ER stress and are precursors for inflammatory mediators.

Until recently, our understanding of how microglial metabolism relates to activation and function has been somewhat extrapolated from the peripheral macrophage literature. However, recent scRNA-seq studies mentioned above have highlighted that microglial metabolism may be similarly altered in response to changing bioenergetics during the development of NDs. For example, several studies have highlighted the need for increased expression of genes associated with lipid metabolism (e.g., TREM2, LPL and ApoE) during development, damage and disease (Keren-Shaul et al., 2017; Hammond et al., 2019). While this suggests a need for increased FAO in these activation states, further studies are needed to empirically define the role of these factors in microglial lipid metabolism and inflammation, particularly in the context of ND. It is important to point out that while the metabolism of microglia may be similar to macrophages in many aspects, it is likely that access to limited metabolic substrates in the CNS (e.g., no very-low-density-lipoproteins [VLDL] or LDL) has led to the development of somewhat distinct metabolic profiles, that have not yet been clearly defined.

## Fatty Acid Metabolism in Microglia

### Overview

The brain’s dry weight is made up of about 50% lipid, most of which is comprised of myelin enriched white matter (Hamilton et al., 2007). Myelin is a specialized OL membrane that contains ∼43% phospholipid (PL), ∼28% glycosphingolipid, and ∼28% cholesterol of its total lipid content (Morell, 1999). PLs are derived from FAs in a biosynthetic pathway that is initiated by fatty acyl-CoA synthase, which combines a FA with acyl-coenzyme A (acyl-CoA) using ATP to create fatty acyl-CoA (Yamashita et al., 2014). Glycerol-3-phosphate acyltransferase (GPAT) adds a glycerol backbone, creating a single-tailed lysophosphatidic acid (LPA) (Yamashita et al., 2014). CoA replenishes it’s acyl group from acyl-carnitine via the carnitine transport system within the mitochondria (Yamashita et al., 2014). Within the ER, LPA is further modulated to give rise to TGs, diacylglycerols (DAGs), and PLs (Yamashita et al., 2014). These PLs are transported through the Golgi, to other organelles, and to the polymorphic cell surface along with proteins, cholesterol, and other FA containing glycolipids (GLs). PLs are important for providing membrane integrity, lipid raft formation, signal transduction, providing curvature to the cell membrane, vesicle formation, apoptosis, and in the production of pro- or anti-inflammatory mediators. Moreover, they are becoming increasingly implicated in the pathophysiology of NDs.

### Phospholipids

Phospholipids constitute 45% of the total dry weight of the brain, and are key to several pathways, such as the synthesis and turnover of neuronal and glial membranes and signaling. Since these processes are critical to CNS health, it is not surprising that there are substantial energy demands associated with PL metabolism, with 5% of the brains ATP being used for the turnover of 1,2-diacyl type PLs (Purdon et al., 2002). A number of reports have suggested that FAs and PLs are increased during pathological conditions such as inflammation, hypoxia and ischemia, and it is likely that this represents release of FAs from brain PLs, or indeed PLs from vital brain structures. For example, in individuals with PD, total serum PL levels are increased without any signs of cognitive impairment and positively correlate with the progression of their cognitive impairment (Li et al., 2015). This finding suggests that increased PL levels could be an early marker for PD onset and disease progression. Furthermore, the enzymes involved in synthesizing phosphatidylserine (PS), phosphatidylcholine (PC) and phosphatidylethanolamine (PE) are increased in the substantia nigra of PD patients (Ross et al., 2001). The increase in total serum PL may indicate cell membrane damage in the substantia nigra, which triggers a compensatory increase in PS, PE, and PC synthesis. Interestingly, while total serum PL is increased, PS 40:4 is decreased (Zhang et al., 2017). In non-apoptotic cells, PS is asymmetrically present within the inner layer of the cell membrane. However, in PD, apoptotic neurons extrovert PS to the surface of the cell membrane, via inactivated flippase and activated scramblase enzymes, to signal phagocytes to remove the cell (Wei et al., 2013). Microglia are known to target these apoptotic cells via expression of PS-specific receptors (Fadok et al., 2000). In recent years several microglia-derived factors that participate in PS-stimulated phagocytosis have been identified. For example, Milk fat globule epidermal growth factor-8 (MFG-E8) is secreted by microglia and links exposed neuronal PS to vitronectin receptors, which modify the actin cytoskeleton to stimulate microglial phagocytosis of dying neurons (Fricker et al., 2012). Therefore, it is plausible that activated microglia may have increased uptake of PS during inflammation, which could potentially drive decreased serum levels observed in PD. It is also plausible that elevated serum PL, could indicate defective microglial phagocytosis.

### PUFAs

Polyunsaturated fatty acids have more than one carbon-carbon double bond within the FA hydrocarbon chain. n-3 PUFAs are characterized by a double bond three atoms away from the ω-methyl group. While mammals cannot synthesize n-3 PUFAs, they are available from the diet in the form of essential FAs, such as alpha-linolenic acid (ALA). n-3 PUFAs are associated with a range of benefits: from preventing age related cognitive decline to preventing metabolic syndrome and improving cardiovascular risk factors (Schwinkendorf et al., 2011; Layé et al., 2015; Trépanier et al., 2016). In contrast, omega-6 (n-6) PUFAs, which have their last double bond 6 atoms away from the terminal methyl group, are generally associated with the worsening of many chronic diseases such as obesity, neuroinflammatory diseases, and asthma (Simopoulos, 2016; Brigham et al., 2019). Within the cell membrane, the ratio of n-3 to n-6 PUFAs can influence cell functions (Hennig and Watkins, 1989; Schwinkendorf et al., 2011; Simopoulos, 2016). Both n-3 and n-6 PUFAs are synthesized using the same elongases and desaturases, so they must compete for their creation and integration into the cell membrane (Simopoulos, 2011). PUFAs are abundant within the CNS, making up 30% of the brain’s FAs; n-3 PUFAs account for a third of these (Hamilton et al., 2007). The distribution of PUFAs isn’t homogenous across lipid class (Simopoulos, 2008). For example, TGs and CEs contain eicosatetraenoic acid (EPA), 20:5, and ALA, whereas ALA is scarcely present in PLs, which contain a high concentration of docosahexaenoic acid (DHA), 22:6, and EPA (Simopoulos, 2008). PUFAs are also heterogeneously located across different brain regions. About 30% of all fatty acids in the outer segment membrane of retinal photoreceptors are n-3 PUFAs (Bazan et al., 1990). Consequently, a diet rich in n-3 PUFAs significantly slows the decline in visual acuity in patients with retinitis pigmentosa (Berson et al., 2012). In addition, the most prevalent n-3 PUFA in the body, DHA, is found at concentrations that are several hundred-fold higher than EPA in the brain and retina (Arterburn et al., 2006). This may be caused by the brain’s preferential usage of EPA as an energy source that is rapidly catabolized via FAO (Chen and Bazinet, 2015). Importantly, DHA cannot be synthesized *de novo* in the brain, and until fairly recently, the mechanism of long chain (LC)-FA transportation across the blood brain barrier (BBB) has remained unknown. However, recent studies have shown that a member of the Major Facilitator Superfamily Domain Containing 2A (Mfsd2a) can transport DHA, in the form of lysophosphatidylcholine (LPC) (Nguyen et al., 2014). In support, Mfsd2a-knockout mice show markedly reduced levels of DHA in the brain, accompanied by neuronal loss and cognitive deficits (Nguyen et al., 2014). Although initially studies report the expression of Mfsd2a largely in the endothelial cell, subsequent studies have also shown that Mfsd2a is present, albeit at a lower level in OLs, OPCs and astrocytes (Chan et al., 2018). Moreover, increased numbers of activated microglia are observed in the subretinal space of Mfsd2a knockout mice compared to wild-type controls (Wong et al., 2016).

Omega-3 and omega-6 PUFA containing PLs bring about dichotomous secondary mediators when turned over at the level of the cell membrane. The phospholipase A_2_ (PLA_2_) superfamily of enzymes, hydrolyze FAs from the sn-2 position of membrane PLs, and contain 15 groups that differ in their ability to recognize and respond to various PL substrates. Among these groups, cytosolic group IV of PLA_2_ (cPLA_2_) releases arachidonic acid (AA), whereas calcium dependent group VI PLA_2_ releases DHA (Jenkins et al., 2004). Typically, free DHA is enzymatically metabolized by cyclooxygenase 2 (COX2) and lipoxygenases (LOXs) into resolvins, protectins, and maresins, which are known to be potent anti-inflammatory specialized pro-resolving mediators (SPMs) (Serhan, 2014; Calder, 2015). Free AA, however, is metabolized into prostaglandins, prostacyclins, thromboxanes, and leukotrienes. With a few exceptions, these AA derived mediators are known to produce potent pro-inflammatory effects (Lukiw and Bazan, 2000). Positron emission tomography (PET) has been used to study AA incorporation into the brain (Robinson et al., 1992; Rapoport, 1999), and made important contributions to our understanding of AA turnover and metabolism in various contexts. For example, PET imaging has revealed that AA uptake is elevated in widespread cortical regions in patients with AD compared to healthy controls, which is consistent with the notion that elevated AA is a marker for neuroinflammation (Esposito et al., 2008).

Within microglia, the downstream effects of n-3 PUFA metabolism has been observed to be beneficial in the context of neuroinflammatory diseases. n-3 PUFA supplementation attenuates activation by deacetylation of the High Mobility Group Box 1/Nuclear Factor kappa-light-chain-enhancer of activated B cells (HMGB1)/(NF-kB) pathway in a traumatic brain injury (TBI) mouse model (Chen et al., 2018). This in turn leads to decreased inflammatory markers and neuroprotective effects following injury (Chen et al., 2018). Although n-6 PUFAs are generally considered pro-inflammatory, this is not the case for all n-6 PUFAs. For example, supplementation with palmitic acid, a 16-C SFA, can activate microglia (Tu et al., 2019). However, supplementation with n-6 linoleic acid (LA) is sufficient to reverse this activation (Tu et al., 2019). In addition to microglial polarization, a growing body of evidence suggests that different FA classes uniquely alter energy metabolism irrespective of activation status (Button et al., 2014; Chausse et al., 2019). In a study with real-time metabolic measurements coupled with lipidomic analysis, both oleate, a monounsaturated FA (MUFA), and palmitate, a SFA, promoted oxidative metabolism whereas LPS increased glycolysis (Chausse et al., 2019). Although both FAs promoted OXPHOS, oleate treatment increased CD36 abundance (an important scavenger receptor and LC-FA transporter), as well as PUFA-containing TGs, while palmitate incorporated more PUFAs into PLs (Chausse et al., 2019). It is likely that SFA-induced incorporation of PUFAs into PLs contributes to inflammation since PUFAs are more readily peroxidized (Chausse et al., 2019). It is also of note that LPS activation can decrease MUFAs while increasing SFA concentrations, both of which can induce a pro-inflammatory effect (Button et al., 2014). Further studies using metabolomic and lipidomic analysis need to be conducted to fully elucidate the metabolic alterations that take place in microglia in response to different FAs.

Omega-3 PUFA supplementation has also been shown to enhance microglial phagocytosis of myelin (Chen et al., 2014). Several studies have demonstrated that increasing the n-3 to n-6 ratio in fat-1 mice, which express an n-3 FA desaturase that converts n-6 PUFAs to n-3 PUFAs, has beneficial results on the inflammatory status of microglia (Ma et al., 2006; Kang, 2007; Zhang et al., 2017). A recent study used the cuprizone-model of demyelination and remyelination to compare fat-1 mice to WT (Siegert et al., 2017). The CNS lipid profile revealed increased remyelination in fat-1 mice as well as increased EPA levels and EPA metabolites, such as 18-hydroxyeicosapentaenoic acid (18-HEPE) (Siegert et al., 2017). As previously stated, EPA has a relatively low quantity in the CNS lipid profile, and is rapidly utilized as a carbon source in FAO (Chen and Bazinet, 2015). Taken together, perhaps the energy provided from FAO of EPA is critical in fueling phagocytosis of myelin and production of secondary mediators that further aid in the process of remyelination. It is also possible that EPA maintains oxidative metabolism and prevents the shift toward glycolysis that occurs during pro-inflammatory and metabolic reprogramming in microglia.

In addition to fueling oxidative metabolism, PUFAs are also agonists for nuclear receptors with important roles in microglial activation and function. For example, the peroxisome proliferator activated receptors (PPARs) are a family of nuclear receptors with key roles in lipid and glucose metabolism, inflammation, and proliferation. All three PPAR isoforms (α, β/δ and γ) are activated by naturally occurring FAs such as AA and EPA. Moreover, the downstream metabolite of AA, 15-deoxy-Δ^12–14^-PGJ_2_ (e.g., 15d-PGJ_2_), was the first reported endogenous ligand for PPAR-γ (Forman et al., 1995; Kliewer et al., 1995). Differences in the concentration required to activate PPAR-γ *in vitro*, and the concentrations naturally present, have called the physiological relevance of 15d-PGJ_2_ into question. Nonetheless, PPAR-γ is expressed in primary microglial cells, and its expression is enhanced following 15d-PGJ_2_ supplementation (Bernardo et al., 2000). Interestingly LPS-stimulated primary microglia synthesize large amounts of 15d-PGJ_2_, which is able to downregulate microglial activation through both PPAR-γ-dependent and independent mechanisms (Bernardo et al., 2003). Since these initial findings, several studies have now shown that natural and synthetic PPAR-γ agonists can down-regulate surface antigens and inflammatory mediators (Fumagalli et al., 2018), while increasing expression of anti-inflammatory factors such as Arginase 1 (ARG1) and IL-4 (Song et al., 2016). It is likely that the pivotal role of PPAR-γ in microglial activation lies with its role in metabolic reprogramming. PPAR-γ signaling directs microglia toward protective functions, such as increased FAO and OXPHOS (Fumagalli et al., 2018). Therefore, PPAR-γ agonists have been extensively studied as potential therapeutics to ameliorate aberrant microglial activation during ND, with mixed results.

### FAs in Oxidative Metabolism

**FIGURE 2:**
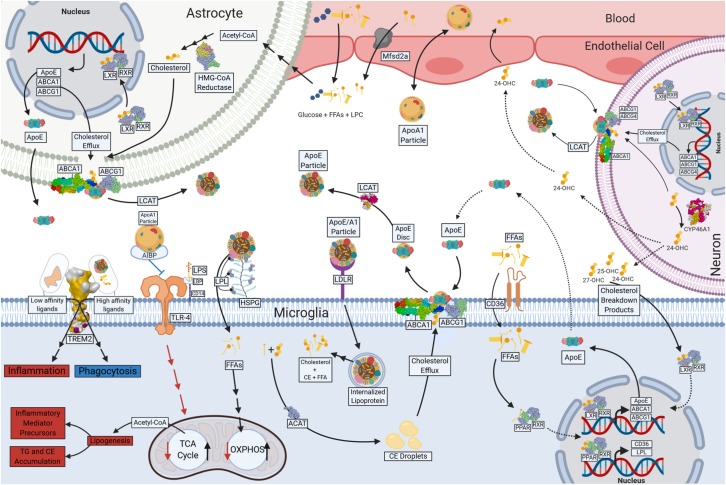
Lipid and Lipoprotein metabolism in microglia. Free fatty acids (FFAs) and glucose cross the blood brain barrier (BBB) and provide energy substrates for neurons and glia. Apolipoprotein A1 (ApoA1) containing lipoproteins traverse the BBB and may play a role in lipid delivery to neurons and glia. Astrocytes are major source of CNS-derived lipoproteins and produce Apolipoprotein E (ApoE) containing lipoprotein particles, which is lipidated following ATP Binding Cassette Subfamily A Member 1 (ABCA1) and ATP Binding Cassette Subfamily G Member 1 (ABCG1)-mediated cholesterol efflux. Astrocyte-derived ApoE particles interact with Triggering Receptor Expressed on Myeloid Cells 2 (TREM2), Toll Like Receptor 4 (TLr-4), Low-Density-Lipoprotein Receptor (LDLR) and Lipoprotein Lipase (LPL) tethered to Heparan sulfate proteoglycans (HSPG) on the cell surface of microglia. Internalized lipoprotein particles or components provide a source of FFAs, phospholipids and cholesterol to microglia, driving oxidative metabolism and anti-inflammatory functions. FFAs act a ligand for Peroxisome Proliferator Activated Receptors (PPARs), which in turn drives transcription of genes involved in cholesterol efflux (ABCG1 and ABCA1) and lipid uptake (LPL and CD36). Phospholipids are used in the generation of cell membranes, and the FAs can be cleaved by Phospholipase A2 (PLA2) resulting in production of downstream metabolites such as Prostaglandins (PGs). In the absence of lipid uptake low affinity ligands bind TREM2, and glycolysis and pro-inflammatory functions such as increased inflammatory mediator production and neutral lipid accumulation (TG and CE). Cholesterol and myelin breakdown products (24-OHC, 25-OHC, and 27-OHC) from neurons also provide ligands for nuclear receptors (Liver X receptor [LXR] and Retinoic acid receptor [RXR]) that regulate ABCA1, ABCG1, and ApoE transcription and the production of microglial derived Lipoproteins. Created with Biorender.com.

Although the brain is only 2% of the total body mass, it utilizes ∼20% of the body’s total ATP. While glucose is the primary source of energy for the brain, it has since been estimated that 20% of the brain’s energy production stems from FAO (Ebert et al., 2003; Bélanger et al., 2011). FAs have also been shown to enter the mammalian brain (Dhopeshwarkar and Mead, 1970; Dhopeshwarkar et al., 1971; Taha et al., 2016). Recent evidence suggests that FA uptake by the brain is both a multifaceted and protein-mediated process (Murphy, 2017). It is likely that technical advances may lead to a deeper understanding of brain metabolism and may challenge the dogma of (largely) exclusive glucose utilization by the brain. In support, several studies have shown that FAs are oxidized in the brain during development and adulthood (Allweis et al., 1966; Warshaw and Terry, 1976). It is believed that FAO occurs predominantly in astrocytes (Ebert et al., 2003). However, since isolated neuronal and glial cells, and mitochondria from both, are able to use FAs to fuel oxidative metabolism (Chausse et al., 2019), it is not implausible that FAs provide an alternative source of energy to neurons and glia, either directly, or via astrocyte-derived lipoproteins (see Figure 2). Thus, a growing body of literature suggests that in addition to the role of lipids as components of myelin debris, or precursors to secondary messengers, FAs may also be an important driver of microglial oxidative metabolism. For example, many genes involved in fatty acid oxidation are expressed in microglia, but this expression is repressed following LPS or INF-γ mediated activation (Mauerer et al., 2009). Importantly, this repression is damped by DHA supplementation (Mauerer et al., 2009).

IFN-β is known to increase FAO and OXPHOS in macrophages (Wu et al., 2016). Although an IFN-β-mediated increase in microglial FAO acid oxidation has not been empirically determined, IFN-β is a well-known therapy for MS, and patients undergoing therapy show in an increase in FAO in peripheral myeloid cells (Croze et al., 2013). Moreover, recent studies have shown that supplementation with the IFN-β in a rat model of AD is sufficient to ameliorate microglial activation, reduce ROS and lipid peroxidation, suggesting that IFN-β helps polarize microglia to a phenotype that favors oxidative metabolism (Mudo et al., 2019). These studies highlight the therapeutic potential of IFN-β and FAO to dampen aberrant microglial activation in ND.

Previously, we have shown microglia lacking LPL have significantly reduced FAO, suggesting that lipid uptake fuels lipid oxidation (Bruce et al., 2018). Conversely, L-carnitine supplementation has been shown to increase FAO and attenuate the LPS induced inflammatory response in microglia leading to improved cognitive and neuronal functions (Gill et al., 2018; Singh et al., 2018). Recent studies have also shown that both oleate and palmitate supplementation can sustain mitochondrial respiration in BV-2 microglia, while LPS treatment diminishes oxidative metabolism in favor of increased glycolysis (Chausse et al., 2019). Since LC-PUFAs are important for microglial function, it is likely that peroxisomal β-oxidation is vital to microglial metabolism. Indeed, studies in mice lacking the multi-functional protein-2 (MFP2), a pivotal enzyme in peroxisomal β-oxidation, become profoundly pro-inflammatory (Beckers et al., 2019). Although further studies are needed to identify the principle components of microglial oxidative metabolism, it is likely that FAO is necessary for physiologic energy production and maintenance of an anti-inflammatory phenotype.

To utilize FAs for energy, they must first be taken up by the cell. FA transport proteins (FATPs), FA binding proteins (FABPs), and scavenger receptors that are categorized as class A (SCARA), B (SCARB), or C (SCARC) among others are utilized in both transportation of esterified and non-esterified FAs across the BBB and the monocytic cell membrane. Importantly, these factors are all expressed in microglia, and are regulated during development and disease. CD36, also known as FA translocase (FAT), is a SCARB that plays a major role in microglial LC-FA uptake, oxidized LDL (oxLDL) and fAβ adhesion as well as activation of the innate immune response and ROS production (Coraci et al., 2002; Doens and Fernández, 2014). Interestingly, CD36 is predominantly expressed in microglia of the cerebellum, which may represent increased requirement for FA substrates in a region of the brain where microglia are relatively sparse and are required to survey larger areas (Grabert et al., 2016). Importantly, FAs are often not transported freely. They are instead transported in the form of esterified FAs and CE within lipoproteins.

## Cholesterol Metabolism in Microglia

Lipoprotein derived CE is broken down by CE hydrolase to produce cholesterol and free FAs (FFAs). Although relatively understudied, recent proteomic analysis of specific cell types in the brain has shown that neutral CE hydrolase 1 is abundant in neurons and glia (Viader et al., 2016). Cholesterol is an essential component of the cell membranes that are especially important within the brain. The CNS contains 23% of the body’s total cholesterol content; most of which is a major cell membrane component of neurons and myelin (Hamilton et al., 2007). Unlike other organs, most of the cholesterol comes not from the liver, but from synthesis within the brain itself. *De novo* cholesterol synthesis is not very efficient within neurons, and is primarily achieved by astrocytes (Nieweg et al., 2009). Synthesis of cholesterol, within astrocytes is regulated by sterol regulatory element binding proteins (SREBPs) as well as 3-hydroxy-3-methyl-glutaryl-coenzyme A reductase (HMGCR) (Ye and DeBose-Boyd, 2011). Cholesterol synthesis is vital in the developing CNS, however, in the healthy adult brain, there is a very low level of cholesterol synthesis (Lavrnja et al., 2017). Cholesterol synthesis is high during development due to increased levels of myelinogenesis (Saher et al., 2005), and as previously stated, cholesterol makes up ∼28% of the total lipid volume of myelin (Morell, 1999). In neuroinflammatory diseases, destruction of myelin leads to a buildup of myelin debris that can stimulate microglial phagocytosis, directly inhibit remyelination, and propagates a pro-inflammatory response (Clarner et al., 2012; Kopper and Gensel, 2018). Cholesterol clearance is an important function of microglia (Lavrnja et al., 2017; Cantuti-Castelvetri et al., 2018). In fact, high concentrations of cholesterol are required for the survival of microglia (Bohlen et al., 2017). Additionally, cholesterol is necessary for phagocytosis as well as cytokine release (Churchward and Todd, 2014). Cholesterol homeostasis within the healthy brain also requires its constant elimination from the CNS compartment (Dietschy and Turley, 2004).

The major mechanism for elimination of cholesterol from the brain is its conversion to 24(S)-hydroxycholesterol (24-OHC) which freely exits neurons and the brain (Lund et al., 1999). This is achieved by cholesterol 24S-hydroxylase (CYP46A1) which is solely expressed in neurons in healthy brain (Lund et al., 1999). In neuroinflammatory disease states such as AD and MS, however, CYP46A1 is also highly expressed in both microglia and infiltrating macrophages (Lavrnja et al., 2017). CYP46A1 has been shown to be neuroprotective in the striatum of the R6/2 murine model of Huntington’s disease (Boussicault et al., 2016). These findings highlight the importance of cholesterol elimination and its therapeutic potential. What happens when cholesterol elimination is inadequate, and cholesterol accumulates in the microenvironment? Importantly, CE accumulation is observed in both familial AD (FAD) and sporadic late onset AD (SAD) (van der Kant et al., 2019). Specifically, CE-containing lipids droplets have been shown to accumulate in the SAD brain (Chan et al., 2012). These findings have also been corroborated with observations of increased CE in mouse models of AD (Yang et al., 2014).

In chronic neuroinflammatory states, myelin debris containing phagocytes become “foamy” due to a buildup of FAs, cholesterol, and their breakdown products (Cantuti-Castelvetri et al., 2018). As previously stated, there is a buildup of 7KC in the CSF of patients with MS (Diestel et al., 2003; Leoni et al., 2017). 7KC metabolism is not well understood, however studies suggest that 7KC degradation is facilitated by either 27-hydroxylase or 11 β-hydroxysteroid dehydrogenase type 1 (11β-HSD1) (Lyons and Brown, 2000; Mitiæ et al., 2013). Therefore, a buildup of 7KC is likely associated with ineffective microglial cholesterol metabolism. For example, MS patients have increased CSF cholesterol, likely as a result impaired cholesterol elimination (van de Kraats et al., 2013). These breakdown products are natural ligands for the liver X receptor (LXR) (see Figure 2). When ligand-bound LXR creates a heterodimer with retinoid X receptor (RXR), which transcriptionally regulates expression of cellular cholesterol efflux proteins such as ApoE and ATP-binding cassette transporters (ABCs) (Zhao and Dahlman-Wright, 2010). ABCA1 and ABCG1 are vital for microglial lipoprotein metabolism as they are responsible for the efflux of cholesterol which gets incorporated into ApoE containing high-density-lipoprotein (HDL)-like particles (Hirsch-Reinshagen et al., 2004). Foamy phagocytes contain increased levels of 24-OHC, 25-OHC, and 27-OHC as well as up-regulated expression of LXR, ABCA1 and ABCG1 (Zhao and Dahlman-Wright, 2010), which may be an attempt to rid foamy microglia or perivascular macrophages of the cholesterol containing myelin debris (Nathanael et al., 2012).

As previously stated, aged microglia have defective phagocytic cholesterol clearance which limits remyelination in MS (Cantuti-Castelvetri et al., 2018). Myelin debris accumulates in the cells’ cytosol as lipid droplets and needle-shaped cholesterol crystals (Cantuti-Castelvetri et al., 2018). Moreover, debris accumulates within microglial lysosomes, leading to lysosomal rupture, and inflammasome stimulation (Cantuti-Castelvetri et al., 2018). Altogether, the balance between cholesterol intake and cholesterol efflux may play a major role in the pathogenesis and progression of neuroinflammatory diseases. Perhaps increased cholesterol efflux may reverse metabolic derangements. For example, a recent study has demonstrated that apolipoprotein A-1 (ApoA-1) binding protein (AIBP) binds to TLR4, a component of the innate immune response, within the cell membranes of activated microglia to stimulate removal of excess cholesterol via efflux to HDL or ApoA-1 (see Figure 2) (Woller et al., 2018). Furthermore, intrathecal AIBP reduces TLR4 dimerization and neuroinflammation in the spinal cord in facilitated pain states (Woller et al., 2018). Although microglial cholesterol efflux is important, a major contributor of cholesterol and oxysterol efflux is achieved by astrocytes and neurons (Chen et al., 2013). Astrocytes also use ABCA1 and ABCG1 for cholesterol efflux into lipoproteins, whereas neurons use ABCA1, ABCG1, and ABCG4 (Chen et al., 2013). Cholesterol efflux seems to have therapeutic potential and can be modulated via lipoprotein metabolism.

## Lipoprotein Metabolism in Microglia

Tightly regulated lipid metabolism in the CNS is critical for maintaining normal brain functions. However, because lipids such as cholesterol and triglycerides are insoluble in water, they are transported with apoproteins, which creates an emulsion that can be transported to other cells via the extracellular milieu and/or peripheral circulation. The resulting lipoproteins are complex particles composed of a central hydrophobic core of neutral lipids such as CEs and TGs, surrounded by an amphipathic monolayer of PLs, free cholesterol, and apolipoproteins.

Although lipoprotein metabolism in the circulation and the CNS vary considerably, it is useful to outline what we have learned from circulating lipoproteins for a clearer understanding of lipoprotein metabolism as a whole. In the peripheral circulation, exogenous lipoprotein metabolism begins with TG-rich chylomicrons (CMs) that are hydrolyzed by LPL to produce CM remnants, which are then taken up into the liver. The liver initiates endogenous lipoprotein production via the formation of VLDL carrying a range of apoproteins including apolipoprotein B-100 (ApoB-100) and ApoE. VLDL is hydrolyzed further by LPL to form intermediate-density-lipoproteins (IDL) and low-density-lipoproteins (LDL), also carrying ApoB-100 and ApoE, which are then bound to LDL receptors and internalized by cells of key metabolic tissues such as muscle, heart, adipose tissue and liver (Grunfeld and Feingold, 2018). There are no reports of VLDL and LDL in the CNS. However, it is tempting to speculate that lower-density-lipoproteins could penetrate the BBB in pathological conditions such as increased inflammation. In addition, it is possible that larger lipoproteins could have enhanced penetrance in the circumventricular organs of the brain; regions devoid of a typical BBB with intimate contact between the blood and CSF such as the median eminence (Gross and Weindl, 1987).

In contrast, HDL or HDL-like lipoproteins may traverse the BBB to enter the CSF under physiologic conditions (Stukas et al., 2014). In the periphery, the first step in HDL formation involves the synthesis of ApoA-I by the liver and intestine. After secretion, ApoA-I acquires cholesterol and PLs that are effluxed by hepatocytes and enterocytes. Cholesterol efflux is regulated by ABCA1, ABCG1 and class B scavenger receptor protein (SR-B1). It is thought that increased intracellular cholesterol leads to oxysterol formation, which triggers the activation of the LXR-RXR heterodimer and ABCA1 and ABCG1 expression in order to efflux/remove cholesterol from the cell. During lipidation, Lecithin–cholesterol acyltransferase (LCAT) transfers a FA from PLs to free cholesterol forming CE, which can then be transferred to the HDL-core (Grunfeld and Feingold, 2018). Sterol O-acyltransferase (ACAT) shares a similar function to LCAT, however it catalyzes the formation of CEs from cholesterol and LC-fatty acyl-CoA and plays a role in hepatic lipoprotein assembly (Li et al., 1999). Although ApoA-I is not synthesized in the brain, fluorescently labeled ApoA-I rapidly accumulates in the CSF after intravenous injection (Stukas et al., 2014), highlighting a plausible role in cholesterol delivery to and from the brain (Mahley, 2016).

ApoA-I is a major apoprotein of HDL-like particles in the brain, second only to ApoE. Unlike ApoA-I, ApoE is synthetized within the CNS predominantly by astrocytes (including specialized astrocytic cells, Bergmann glia, tanycytes, pituicytes, and retinal Muller cells) (Boyles et al., 1985). It has been suggested that astrocytes secrete cholesterol-rich ApoE/HDL through an interaction with ABCA1, to provide cholesterol to other cells of the CNS (Ito et al., 2014). Astrocyte derived ApoE/HDL cholesterol is involved in the provision of lipids for axonal growth and regeneration (Vance et al., 2000), and synaptogenesis (Mauch et al., 2001). Importantly, other cell types can increase ApoE production when the need for lipid substrates is greater, such as during development, injury or stress. For example, in mice expressing enhanced GFP (EGFP) ApoE, injured neurons produce high levels of EGFP and ApoE mRNA in the hippocampus (Xu et al., 2006). ApoE is also expressed in type 1 NSCs (Hong et al., 2016), and can directly regulate proliferation and spine density (Hong et al., 2016). Similarly, scRNA-seq studies have shown that microglia express higher levels of ApoE in the embryonic mouse brain (E14.5) (Hammond et al., 2019), during LPC mediated demyelination injury (Hammond et al., 2019), and in the late stages of disease pathogenesis in murine models of AD (Keren-Shaul et al., 2017). Although the precise role of increased ApoE production in microglia is unknown and may be activation-state and/or microglial subpopulation specific, it is likely that increased ApoE may be a response to increased intracellular cholesterol accumulation. For example, during late embryogenesis a significant number of OLs undergo apoptosis (discussed above). In addition to microglial phagocytosis of dying cells and myelin debris, resident microglia may also increase ApoE production concurrently in order to efflux accumulating cholesterols and oxysterols in an attempt to maintain normal microglial function. Although this has not been empirically determined, aging microglia show reduced ApoE production and massive cholesterol accumulation, with major detriment to their phagocytic capacity (Cantuti-Castelvetri et al., 2018). Interestingly, LXR agonists have been shown to reduce neurodegeneration and pathology in neurogenerative animal models, suggesting that ABCA1/ABCG1-mediated cholesterol efflux promotes homeostatic glial functions (Cantuti-Castelvetri et al., 2018).

In addition to cholesterol efflux, apoproteins and lipoproteins have been repeatedly implicated in microglial phagocytosis. For example, ApoE is known to bind to LDL receptors (e.g. LDL receptor [LDLR], LDL Receptor Related Protein 1 [LRP1] and VLDL receptor [VLDLR]) on the surface of microglia and become internalized via receptor-mediated endocytosis (Pocivavsek et al., 2009). Recently, several other cell surface factors that may participate in lipoprotein uptake and/or endocytosis have been identified. TREM2 is a recently discovered AD risk gene that encodes a single-transmembrane protein that is selectively expressed in the microglia in the CNS (Klesney-Tait et al., 2006). Although the physiological role of TREM2 is still under investigation, it is thought to be protective in the context of AD since TREM2 deficiency in the 5XFAD mouse model causes increased amyloid deposition (Wang et al., 2015), whereas overexpression of TREM2 in the Amyloid precursor protein/Presenilin-1 (APP)/(PS1) mouse ameliorates neuropathology (Jiang et al., 2014). Since Aβ is known to bind to ApoE, the protection offered by TREM2 maybe conferred through its ability to bind to phospholipids and apoproteins (ApoE and apolipoprotein J [ApoJ]) and to increase phagocytosis (Atagi et al., 2015). TREM2 has been shown to interact with Aβ-bound lipoproteins to facilitate Aβ uptake by microglia and human macrophages (Yeh et al., 2016). In fact, TREM2 serves as an extracellular lipid and lipoprotein receptor that modulates the inflammatory profile of microglia depending on the affinity of ligand binding (Lessard et al., 2018). Specifically, high avidity ligands, such as lipids and ApoE, bind TREM2 causing phosphorylation of two tyrosine residues within the adjacent transmembrane DNA polymerase III-Activation Protein 12 (DAP12) (Lessard et al., 2018). This initiates a cellular cascade culminating in survival, proliferation, phagocytosis, and motility depending on the ligand (Lessard et al., 2018). Conversely, when TREM2 is associated with low avidity ligands, an intracellular cascade is initiated that may promote inflammatory functions within the cell (Lessard et al., 2018). Recent work suggests that PS and PE from apoptotic neurons (which are not exposed on the surface of healthy cells) act as signals for TREM2-mediated microglial activation (Shirotani et al., 2019). This topic has been more comprehensively reviewed in a number of recent peer-reviewed articles (Jay et al., 2017; Gratuze et al., 2018).

Human ApoE exists as three major isoforms ApoE2, ApoE3 and ApoE4, which are encoded by three allelic variants at a single gene locus on the long arm of chromosome 19. Each isoform differs by 2 amino acid substitutions (ApoE2 [Cys112, Cys158], ApoE3 [Cys112, Arg158], and ApoE4 [Arg112, Arg158]), which significantly alters their lipid and receptor binding affinities (Mahley and Rall, 2000; Wu and Zhao, 2016). ApoE2 has a lower affinity for LDLRs compared to ApoE3 and ApoE4. In addition, ApoE2 and ApoE3 preferentially bind to small, PL-rich HDL whereas ApoE4 preferentially binds to larger, TG-rich lipoproteins (Wu and Zhao, 2016). ApoE3 is present in approximately 75% of the population and is believed to play a neutral role in AD. In contrast, ApoE2 is relatively rare (5% incidence) and is considered to be protective against AD (Wu and Zhao, 2016). While the mechanism of this protection is unclear, it is plausible that its differential association with CNS derived HDL-like lipoprotein particles plays a major role. Importantly, the ApoE4 isoform (present in 20% of the population), is present in nearly 50% of AD patients (Wu and Zhao, 2016). Although there has been a major research effort attempting to understand the increased risk of AD conferred by ApoE4, the mechanisms remain elusive. Nonetheless, in murine models of AD, it has been established that ApoE isoforms differentially effect Aβ accumulation in a dose- and isoform- dependent manner, with hippocampal Aβ burden: ApoE2 < ApoE3 < ApoE4 (Castellano et al., 2011). Findings from *in vitro* studies have shown that ApoE4 can inhibit TREM2 expression in primary murine microglia (Li et al., 2015), therefore potentially blocking the appropriate microglial response to Aβ accumulation.

ApoE4 is not the only apoprotein associated with increased AD risk. In 2009, two Genome Wide Association Studies (GWAS) identified ApoJ (otherwise known as Clusterin [CLU]) as a novel SAD-risk gene (Harold et al., 2009; Lambert et al., 2009). ApoJ is now considered the third greatest risk factor for SAD. Although a number of possible mechanisms have been proposed, including its role in TREM2-mediated microglial lipoprotein and Aβ clearance (Yeh et al., 2016), its precise function is has yet to be determined (Foster et al., 2019).

## The Role of Lpl in Microglia Function and Metabolism

In the periphery, LPL is primarily involved in the hydrolysis of CM-TG and VLDL-TG. In the murine brain LPL is predominantly expressed in microglia/macrophages and the OPC (Zhang et al., 2014), whereas in the human brain LPL is predominantly expressed in microglia (Zhang et al., 2016). Although the role of LPL in microglia is not well understood, LPL has been repeatedly implicated in AD pathogenesis in humans. For example, patients with AD have reduced LPL immunoreactivity in the dentate gyrus and reduced LPL activity in their CSF (Gong et al., 2013). In addition, loss of function LPL polymorphisms have been linked to reduced enzymatic activity, increased VLDL-TG, and increased AD risk (Ren and Ren, 2016). In contrast, patients with a gain-of-function LPL polymorphism (447Ter) have increased LPL activity concomitant with lower VLDL-TG, higher HDL, and significantly reduced hippocampal amyloid plaque formation (Baum et al., 2000). Moreover, there is a growing body of literature highlighting the importance of LPL in microglial function. For example, in the 5XFAD mouse model of AD, LPL expression is dramatically increased in DAM (Keren-Shaul et al., 2017). Importantly, markedly enhanced LPL expression repeatedly observed in microglial subpopulations, particularly in the context of development (Hammond et al., 2019), demyelination (Hammond et al., 2019), and disease (Mathys et al., 2017). In both mouse and human brains, LPL co-localizes with microglia that have internalized Aβ (Keren-Shaul et al., 2017), suggesting that microglial-LPL may also play a role in Aβ uptake. Indeed, LPL has been shown to directly bind Aβ and play a role in uptake and degradation in astrocytes (Nishitsuji et al., 2011). However, exactly how LPL may interact with Aβ remains to be determined. Since LPL is known to directly interact with LRP1 (Beisiegel et al., 1991; Chappell et al., 1992), which is major receptor for both ApoE and Aβ (Herz and Strickland, 2001), it is plausible that LPL could facilitate Aβ uptake through interaction with other factors expressed on the microglia cell surface. LPL also directly interacts with ApoE (Baum et al., 2000), and it is plausible that the strength of the interaction may vary depending on the isoform’s specific lipid binding avidity. The role of LPL in microglia could conceivably link both lipid metabolism and inflammation. We have previously shown that the BV-2 microglia is polarized to a robust inflammatory phenotype following LPL loss, a phenotype that is recapitulated in primary microglia (Bruce et al., 2018). Moreover, loss of LPL shifts the metabolic profile of microglial cell lines toward increased glycolysis and reduced oxidative metabolism, reminiscent of the Warburg-like metabolism observed in inflammatory macrophages (Bruce et al., 2018). Our findings suggest that the importance of LPL in microglia may extend beyond phagocytosis of Aβ to regulation of metabolic and inflammatory phenotype.

## Future Strategies

The findings highlighted in this review suggest that lipid and lipoprotein metabolism is a tightly regulated component of microglial immunometabolism. The changes seen in microglial lipid metabolism during damage and disease are some of the most drastic and profound responses that have been observed to date. This suggests that microglial lipid, cholesterol, and lipoprotein metabolism may be novel therapeutic targets for the treatment of CNS disorders such as ND. Since pro-inflammatory microglia and perivascular macrophages that may contribute to ND progression exhibit increased glycolysis but decreased OXPHOS and FAO, it is plausible that modulating substrate utilization in microglia could ameliorate neuroinflammation. Although the optimal method to switch substrate usage in microglia has not been defined, several studies have shown that dietary approaches are sufficient to modulate microglial immunometabolism. Ketogenic diets typically contain very low carbohydrate, but very high fat levels and therefore have the capacity to promote FAO and limit glycolytic flux. In a recent study using a murine model of glaucoma, a ketogenic diet (10.4% protein, 0.1% carbohydrate, and 89.5% fat) was able to reduce markers of microglial activation such as Ionized calcium binding adaptor molecule 1 (Iba1) (Harun-Or-Rashid and Inman, 2018). It has also been shown that ketones can directly modulate inflammation in microglia. For example, β-hydroxybutyrate activates G-protein-coupled receptors 109A (GPR109A) to attenuate NF-κB signaling and pro-inflammatory cytokine production (Fu et al., 2015). These studies suggest that ketogenic diets may be a promising strategy; however, it is important that we do not halt the waves of microglial activation that may be potentially protective in the contexts of certain disease states. In addition, it is critical to carefully evaluate the lipid composition of these diets, since only those including LC-FAs would promote mitochondrial oxidative metabolism. It is also important to note that ketogenic diets may not be optimal for overall metabolic health, and other strategies, such as caloric restriction, and fasting may increase ketone delivery to the brain to down-regulate microglial activation without markedly elevating lipid consumption (Loncarevic-Vasiljkovic et al., 2012; Tu et al., 2012; Ghosh et al., 2018).

## Concluding Remarks

In summary, microglia are dynamic cells that are not only critical for homeostatic brain functions but in mitigating the response to a variety of stimuli such as primary myelination, and demyelination. During their response, microglia tightly regulate lipid and lipoprotein metabolism in order to fuel greater bioenergetics needs, to phagocytose and process lipid-rich debris, and to produce precursors for secondary messengers. Although recent studies have galvanized the importance of lipid and lipoprotein metabolism in microglia, this emerging field is likely to reveal further important mechanisms going forward. In addition, further study into the role of lipid metabolism in the polarization of microglial inflammatory status may highlight novel approaches that modulate metabolism to ameliorate neuroinflammatory and NDs.

## Author Contributions

KB devised the framework of the manuscript. BL designed and created the figures. BL and KB shared equal parts in drafting and editing the manuscript.

## Conflict of Interest

The authors declare that the research was conducted in the absence of any commercial or financial relationships that could be construed as a potential conflict of interest.

## Abbreviations

11β-HSD 1: 11β-hydroxysteroid dehydrogenase type 1
15d-PGJ_2_: 15-deoxy- Δ ^12–14^-PGJ_2_
18-HEPE: 18-hydroxyeicosapentaenoic acid
24-OHC: 24(S)-hydroxycholesterol
5XFAD: 5 familial AD mutations
7KC: 7-ketocholesterol
A β: amyloid beta
AA: arachidonic acid
ABC: ATP-binding cassette transporter
ACAT: Sterol O-acyltransferase
acyl-CoA: acyl-coenzyme A
AD: Alzheimer’s disease
AIBP: ApoA-1 binding protein
Akt: Protein kinase B
ALA: alpha-linolenic acid
ApoA-1: apolipoprotein A-1
ApoB-100: apolipoprotein B-100
ApoE: Apolipoprotein E
ApoJ: apolipoprotein J
APP: Amyloid precursor protein
ARG1: Arginase 1
ATGL: Adipose triglyceride lipase
ATP: adenosine triphosphate
BAM: Border-Associated Microglia
BBB: blood brain barrier
CD11b/CD18: C3 receptor
CE: cholesterol ester
CLEM: Correlative light and electron microscopy
CLU: Clusterin
CM: chylomicron
CNS: central nervous system
COX2: cyclooxygenase 2
cPLA2: cytosolic group IV of PLA2
CPT1A: Carnitine Palmitoyltransferase 1A
CSF: cerebrospinal fluid
CSF-1R: Colony stimulating factor 1 receptor
CX3CL1: chemokine fractalkine
CX3CR1: C-X 3-C Motif Chemokine Receptor 1
CXCL1: chemokine (C-X-C motif) ligand 1
CYP46A1: cholesterol 24S-hydroxylase
DAG: diacylglycerol
DAM: disease associated microglia
DAP12: DNA polymerase III-Activation Protein 12
db/db: leptin deficient mouse model of type 2 diabetes
DHA: docosahexaenoic acid
EGFP: enhanced GFP
EPA: eicosatetraenoic acid
ER: endoplasmic reticulum
fA β: fibrillar A β
FA: fatty acid
FABP: FA binding protein
FAD: familial AD
FAO: FA oxidation
FATP: FA transport protein
FFA: free FA
GFP: green fluorescent protein
GL: glycolipid
GPAT: Glycerol-3-phosphate acyltransferase
GPR109A: G-protein -coupled receptors 109A.
GWAS: Genome Wide Association Studies
HDL: high-density lipoprotein
HFD: high-fat diet
HMGB1: High Mobility Group Box 1
HMGCR: 3-hydroxy-3-methyl-glutaryl-coenzyme A reductase
HSL: hormone sensitive lipase
Iba1: Ionized calcium binding adaptor molecule 1
IDL: intermediate-density-lipoproteins
IFN: interferon
IGF1: Insulin growth factor 1
IHC: immunohistochemistry
iMG: induced microglia-like cell culture
IP: intraperitoneal
LA: linoleic acid
LC: long chain
LCAT: Lecithin–cholesterol acyltransferase
LDL: low-density lipoprotein
LDLR: LDL receptor
LOX: lipoxygenase
LPA: lysophosphatidic acid
LPC: lysophosphatidylcholine
LPL: Lipoprotein Lipase
LPS: lipopolysaccharide
LRP1: LDL Receptor Related Protein 1
LXR: liver X receptor
mA β: monomeric A β
MFG-E8: Milk fat globule epidermal growth factor-8
MFP2: multi-functional protein-2
Mfsd2a: Major Facilitator Superfamily Domain Containing 2A
MS: multiple sclerosis
MS4A7: Membrane-spanning 4-domains subfamily A member 7
mTOR: mammalian target of rapamycin
MUFA: monounsaturated FA
n-3: omega-3
n-6: omega-6
ND: neurodegenerative disease
NF-kB: Nuclear Factor kappa-light-chain-enhancer of activated B cells
NPC: neural progenitor cell
NSC: neural stem cell
oA β: oligomeric A β
OL: oligodendrocyte
OPC: oligodendrocyte precursor cell
oxLDL: oxidized LDL
OXPHOS: oxidative phosphorylation
PAM: proliferative-region-associated-microglia
PARP: poly (ADP-ribose)-polymerase
PC: phosphatidylcholine
PD: Parkinson’s disease
PE: phosphatidylethanolamine
PET: Positron emission tomography
PI3K: Phosphoinositide 3-kinase
PL: phospholipid
PLA2: phospholipase A2
PPAR: peroxisome proliferator activated receptor
PPP: pentose phosphate pathway
PS: phosphatidylserine
PS1: Presenilin-1
PSD95: postsynaptic density protein 95
PUFA: polyunsaturated FA
ROS: reactive oxygen species
RXR: retinoid X receptor
SAD: sporadic late onset AD
SCARA: class A scavenger receptor gene
SCARB: class B scavenger receptor gene
SCARC: class C scavenger receptor gene
FAT: FA translocase
scRNAseq: single-cell RNA-sequencing
SFA: saturated fatty acid
siglec-11: sialic acid-binding Ig-like lectin
Slc2a5: Solute carrier family 2 member 5
SNAP25: Synaptosome Associated Protein 25
SPM: specialized pro-resolving mediator
Spp1: Secreted phosphoprotein 1
SR-B1: class B scavenger receptor protein 1
SREBP: sterol regulatory element binding proteins
STED: stimulated emission depletion
SZ: schizophrenia
TBI: traumatic brain injury
TG: triglyceride
TGF β: Transforming growth factor β
TLR: toll-like receptor
Tmem119: Transmembrane Protein 119
TREM2: Triggering Receptor Expressed on Myeloid Cells 2
VLDL: very-low-density-lipoprotein
VLDLR: VLDL receptor.
